# Manual of Chemistry

**Published:** 1895-12

**Authors:** 


MANUAL OF CHEMISTRY. A Guide to Lectures and Laboratory Work for Beginners in Chemistry. A Text-book specially adapted for Students of Pharmacy and Medicine. By W. W. Simon, Ph. D., M. D., Professor of Chemistry and Toxicology, College of Physicians and Surgeons, Baltimore; Professor of Chemistry in the Maryland College of Pharmacy. New (fifth) edition. In one octavo volume of 502 pages, with forty-four engravings and eight colored plates, illustrating sixty-four of the most important chemical tests. Cloth, $3.25. Philadelphia: Lea Brothers & Co. 1895.

This manual, which is especially intended for the use of medical students, shows evidence of its popularity in the production of five editions in a comparatively short space of time.

The first fifty pages of the work go over the theory of chemistry from the elementary definitions to a discussion of the periodic law. While the space devoted to these things is small, still they are very well given. The work, furthermore, treats of descriptive chemistry, qualitative and quantitative analysis, physiological chemistry and the chemicals mentioned in the U. S. Pharmacopeia. This subject matter, though briefly considered in some cases, is very well given indeed.

The book is very well bound and printed and the colored plates are excellent. J. A. M.

